# Role of Exosomes in the Invasion and Metastasis of Ovarian Cancer and Application Potential of Clinical Diagnosis and Treatment

**DOI:** 10.7150/jca.83663

**Published:** 2023-04-17

**Authors:** Yifei Zhu, Cheng Wang, Ziyu Ma, Feifei Li, Chunwei Xu, Weiwei Pan, Aijun Liu

**Affiliations:** 1 Department of Pathology, School of Basic Medical Science, Weifang Medical University, Weifang 261053, China.; 2 Department of Pathology, Seventh Medical Center, General Hospital of PLA, Beijing 100700, China.; 3 Institute of Cancer and Basic Medicine (ICBM), Chinese Academy of Sciences, Hangzhou 310022, China.; 4 Department of Cell biology, College of Medicine, Jiaxing University, Jiaxing 314001, China.

**Keywords:** Ovarian cancer, exosome, cancer therapy, metastasis, drug carrier

## Abstract

Ovarian cancer is a highly lethal form of cancer in females, largely due to extensive metastases that often accompany the initial diagnosis. Exosomes are microvesicles size from 30 to 100nm, which can be secreted by most cells. These special extracellular vesicles play a vital role in the metastasis of ovarian cancer. In this study, we conducted a comprehensive review of current research pertaining to the role of exosomes in ovarian cancer, utilizing the PubMed^®^ and Web of Science databases. Our review highlights the progress in elucidating the mechanisms by which exosomes facilitate ovarian cancer progression. Additionally, we discuss the potential of exosomes as a novel therapeutic target for ovarian cancer treatment. Overall, our review provides valuable insights into the current state of research on exosomes in ovarian cancer therapy.

## Introduction

Worldwide, ovarian cancer accounts for the top five of cancer-related mortality in women. About 80% of cases are diagnosed at advanced international federation of gynecology and obstetrics (FIGO) stages (Ⅲ, Ⅳ) with poor prognosis. In 2021, The American Cancer Society estimated 21,410 new cases of ovarian cancer diagnosed and 13,770 death^1^. Current diagnostic methods for monitoring ovarian cancer include pelvic examination, transvaginal ultrasound, and the serological marker CA125 (carbohydrate antigen 125). However, these methods are not sufficient for early diagnosis of ovarian cancer due to the limitation in both sensitivity and specificity^2^. It has generally been accepted that ovarian cancer cells migrate directly into the peritoneal cavity and omentum through the peritoneal fluid. In addition, epithelial ovarian cancer (EOC) can also spread through blood-borne metastatic routes^3^.

Many studies have shown that cell-to-cell communication in the tumor microenvironment passes through extracellular vesicles (EVs), mainly by a particular type of EVs called exosomes^4^-^6^. These special EVs carry various biomolecules, including proteins, glycans, lipids, metabolites, RNA and DNA^4^. Multiple evidences point out that exosomes play an important role in the metastasis of ovarian cancer by promoting ovarian cancer cell adhesion, invasion, angiogenesis, facilitating immune evasion and chemoresistance and are the main mediators of tumor-stroma crosstalk^7^-^10^. Tumor-derived exosomes can support tumor cell survival and promote metastasis by reprogramming or educating other cells^8^, ^11^. It has been shown that ovarian cancer metastasis is frequently facilitated by the interaction of exosomes with the tumor microenvironment^12^-^14^. Release of exosomal contents can modulate the fate of recipient cells. MicroRNAs (miRNAs) and non-coding RNAs are the main RNA species transported by exosomes, and in addition, mRNA, rRNA and tRNA have also been reported in exosomes^15^. In this review, we summarize recent advances on the role of exosomes in ovarian cancer metastasis and the potential applications of exosomes in diagnosis and treatment of ovarian cancer.

## Role of exosomes in the invasion and metastasis of ovarian cancer

**Figure 1:**
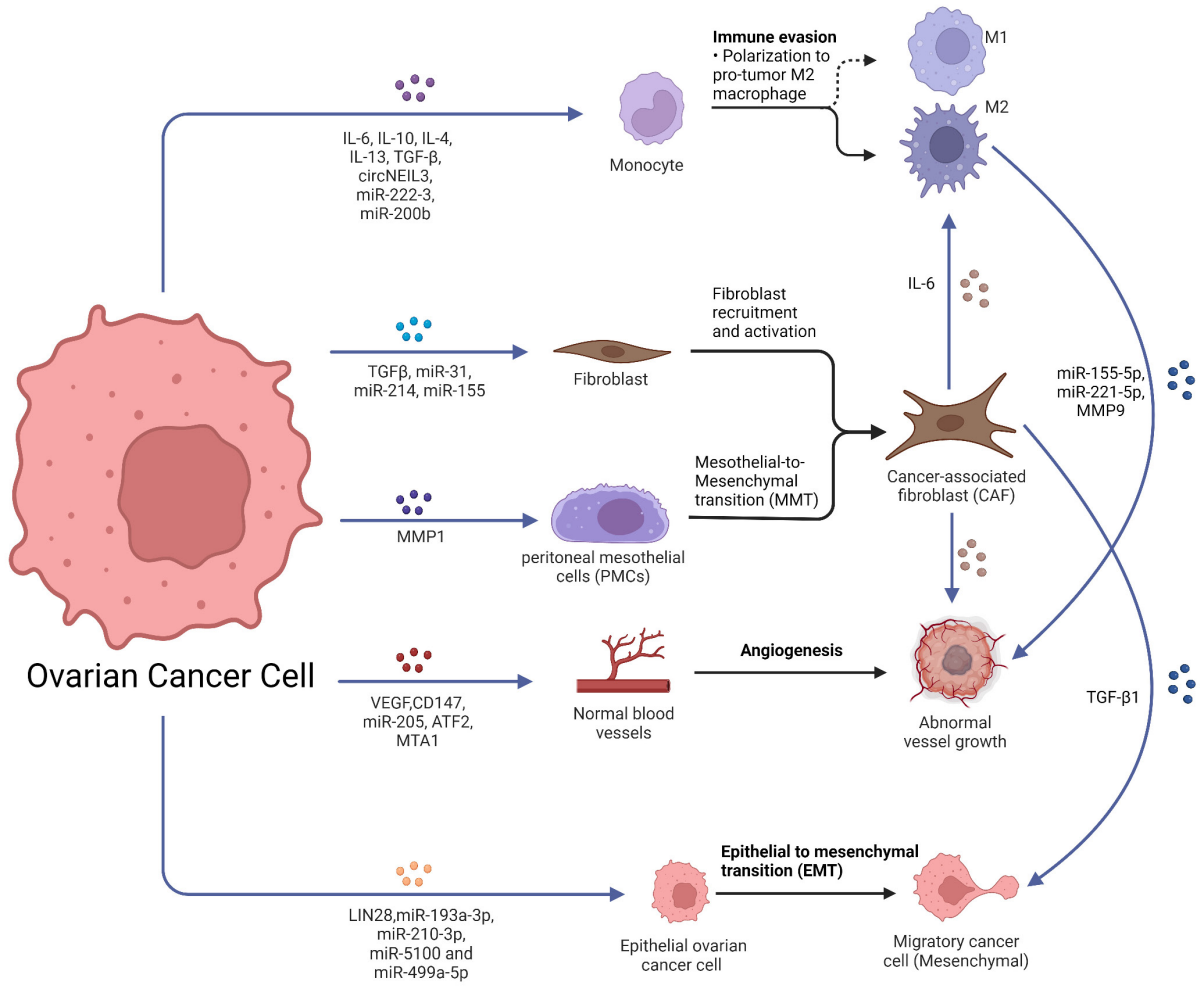
Role of exosomes in ovarian cancer metastasis. Ovarian cancer-derived exosomes with their cargoes promote TAMs polarization, fibroblast recruitment and activation and transfer normal fibroblasts and PMCs into CAFs. Ovarian cancer-derived exosomes promote angiogenesis and EMT. Furthermore, CAF-derived exosomes also promote TAMs polarization, angiogenesis and EMT. TAMs-derived exosomes promoted the malignant phenotype of EOC. Created with BioRender.com.

Unlike other tumors, which typically metastasize through hematogenous or lymphatic pathways, ovarian cancer cells usually metastasize in the peritoneal cavity and greater omentum in the initial stages by some passive mechanism. Much evidence suggests that ovarian cancer metastasis is closely linked to intercellular communication in the tumor microenvironment, and these links can be achieved by soluble factors and EVs (Fig. 1).

### Pre-metastatic niche

The crosstalk between tumor cells and stroma is a crucial feature of tumor progression and metastasis. Pre-metastatic niches (PMNs) are formed by changes occurring in distant organs that prepare for colonization and growth of circulating tumor cells (CTCs), leading to the formation of distant metastases 16. Notably, in addition to intercellular communication in the microenvironment of the primary tumor, tumor can transmit signals to distant organs to promote the formation of PMNs. In PMN models, organs colonized at the time of tumor metastasis are predetermined by the initiation and coordination of primary tumor-secreted factors 17.

**Figure 2:**
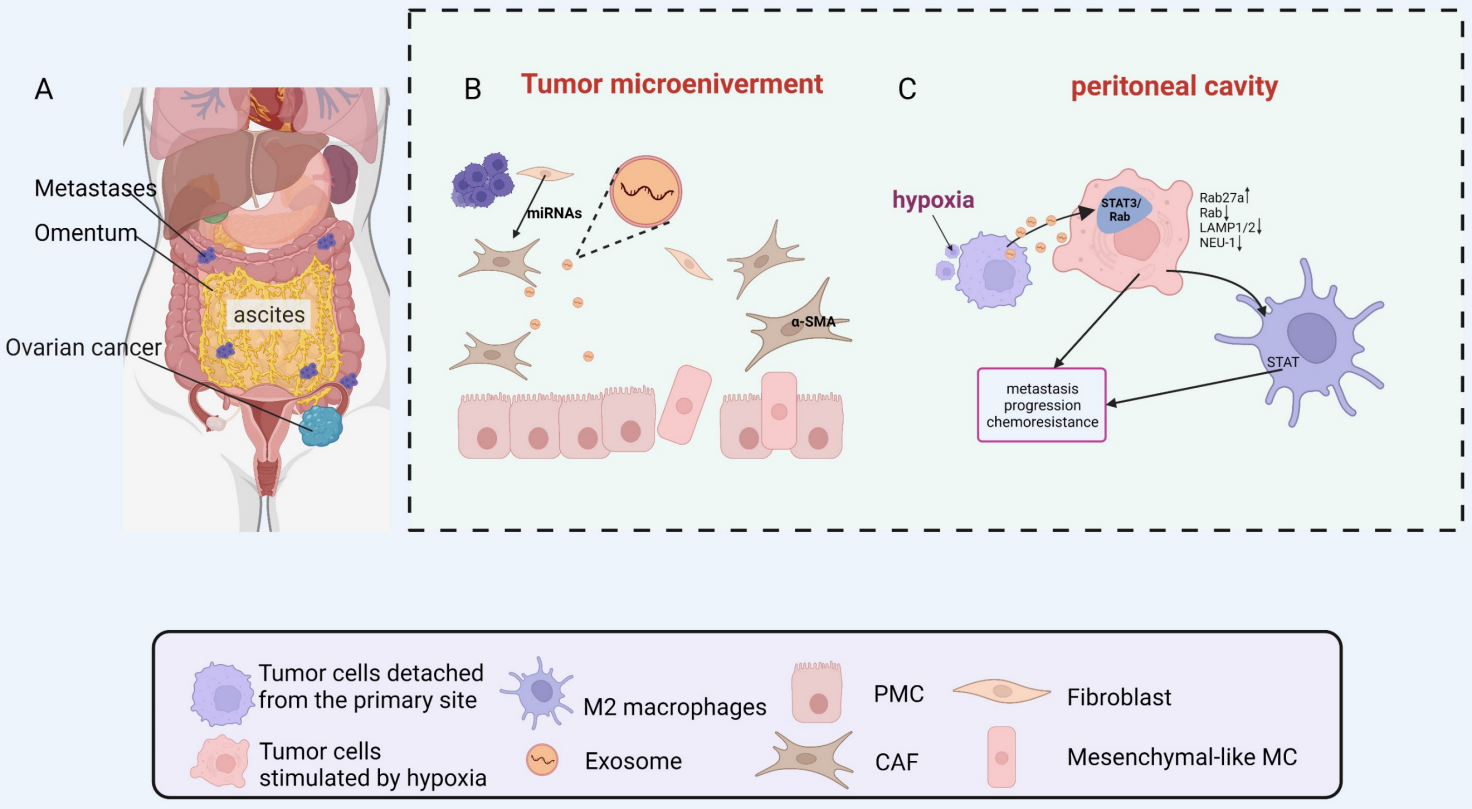
Metastasis of ovarian cancer to the peritoneal cavity through malignant ascites. A. Metastases distributed in the peritoneal cavity. B. MiRNAs secreted by ovarian cancer cells were taken in the PMCs, and PMCs were converted into CAFs. C. The hypoxic ovarian cancer cells deliver exosomes to induce the polarization of M2 macrophages and promote a more aggressive phenotype of cancer cell. Abbreviations: PMC: peritoneal mesothelial cells; CAF: cancer-associated fibroblast; STAT: Signal transducer and activator of transcription; MC: mesothelial cell; miRNA: microRNA. Created with BioRender.com.

The primary tumor secretes cytokines and exosomes which target specific organs, inducing their changes to create a hospitable environment for CTCs (Fig. 2A) 18. Evidence suggests that ovarian cancer-derived exosomes can change PMNs by promoting angiogenesis, immunosuppression, stromal remodeling and transition of cancer-associated fibroblasts (CAFs), helping ovarian cancer to thrive in new environments 19-22.

### Exosomes mediate epithelial-mesenchymal transition

Epithelial-mesenchymal transition (EMT) is a process characterized by loss of polarity of the epithelium and transformation into mesenchymal cells, a phenomenon observed early in tumor invasion and metastasis 23. MiRNAs such as miR-193a-3p, miR-210-3p, miR-5100 and miR-499a-5p can be transmitted via exosomes to help promote tumor metastasis and EMT 24, 25. Exosomes secreted by ovarian cancer cells are also involved in EMT. LIN28, a proto-oncogene secreted in exosomes derived from ovarian cancer cell line IGROV1, has been shown to promote ovarian cancer cells migration and invasion, increasing the expression of EMT-related genes such as ZEB1, NOTCH1, WNT5A, NODAL and SNAI2^26^, ^27^. Additionally, transforming growth factor beta Ⅰ (TGF-β Ⅰ) secreted by CAF-derived exosomes has been demonstrated to induce EMT in ovarian cancer cells in vitro^28^. However, further validation is needed through in vivo experiments.

### The relationship between cancer-associated fibroblasts and exosomes

CAFs are activated fibroblast subsets that play an essential role in the progression and metastasis of various tumors^22^, ^29^. Tumor cells induce CAFs formation through the TGF-β/SMAD pathway, whereby free TGF-β secreted by tumor cells binds to TGF-β Ⅰ and Ⅱ receptors in fibroblasts, prompting downstream SMAD2 and SMAD3 phosphorylation and further forming a complex with SMAD4, resulting in up-regulation of α-SMA transcription^30^. Furthermore, tumor-derived exosomes carry many miRNAs that influence gene transcription by being taken up by fibroblasts and prompt the differentiation of fibroblast into CAFs^5^, ^31^. Several studies have already reported the role of exosomes in tumor stromal cell activation^32^-^34^, yet the mechanism by which ovarian cancer cell-derived exosomes convert normal fibroblasts into CAFs has been less investigated. For example, exosomes derived from ovarian cancer cell line SKOV3 were found to significantly promote proliferation and motility of normal fibroblasts, as well as increase expression of typical markers for CAFs such as α-SMA and tissue inhibitor of metalloproteinase 2 (TIMP2) 35. Additionally, peritoneal mesothelial cells (PMCs) can be converted into CAFs through mesothelial-to-mesenchymal transition (MMT) processes during ovarian cancer peritoneal metastasis (Fig. 2B). Omentum mesothelial cells (MCs) incubated with SKOV3 cell conditioned medium acquired a spindle morphology with increased expression of MMT markers such as α-SMA, fibronectin, collagen Ⅰ, vascular endothelial growth factor (VEGF) and TGF-β Ⅰ 36, indicating accumulation of large numbers of CAFs from PMCs via MMT processes is essential for tumor peritoneal metastasis 36-38. During the development of MMT, exosomes participate in adhesion between tumor cells and MCs. They also facilitate the co-invasion of PMC derived CAFs and tumor cells into the peritoneal matrix 39.

CAF-derived exosomes have been found to be involved in the metastasis of tumor cells and immune escape^40^-^42^. Yan et al. discovered that exosomes derived from CAFs in metastatic ovarian cancer tissues had significantly higher levels of TGF-β Ⅰ compared to exosomes from fibroblasts in normal omental tissue. This increased TGF-β Ⅰ was found to promote the process of EMT in ovarian cancer cells 42. Moreover, these exosomes can induce the transformation of normal fibroblasts into CAFs by modulating the expression of miRNA-31, miRNA-214, and miRNA-155, which are differentially expressed between normal fibroblasts and ovarian cancer-associated fibroblasts^43^.

### Exosomes promote angiogenesis

Tumor-derived exosomes have been found to be involved in the process of angiogenesis, which is essential for tumor progression and metastasis^10^, ^44^. These exosomes are known to contain proangiogenic proteins such as angiogenin, VEGF, interleukin-6 (IL-6), and interleukin-8 (IL-8)^45^, ^46^, as well as encapsulate angiogenic factors which can be translocated to endothelial cells^47^, ^48^. As an example, exosomes highly expressing miR-205 secreted by ovarian cancer cells can promote tumor metastasis by inducing angiogenesis via the PTEN-AKT pathway^11^. Similarly, miR-130a has been found to be involved in promoting angiogenesis by chemoresistant ovarian cancer cell secreted exosomes 47. The activation of transcription factor 2 (ATF2) and metastasis-associated protein 1 (MTA1) upregulated angiogenesis in ovarian cancer-derived exosomes^49^, indicating the vital role of these vesicles in tumor angiogenesis.

In addition to tumor-derived exosomes, those secreted by tumor-associated macrophages (TAMs) and CAFs have also been found to be involved in the promotion of angiogenesis and metastasis^50^-^52^. For instance, M2 macrophage-derived exosomal miR-155-5p and miR-221-5p have been reported to induce angiogenesis of pancreatic ductal adenocarcinoma in an E2F2 dependent manner^53^. Although there are few relevant reports on ovarian cancer, it is likely that these vesicles may also play a role in ovarian cancer progression.

### Relationship between exosomes and TAMs

In contrast to other solid tumors, ovarian cancers in advanced FIGO stage are often characterized by a unique tumor microenvironment termed "malignant ascites", which contains a variety of components including single floating cells or multicellular spheroids, immune cells, fibroblasts, adipocytes, MCs, EVs, cytokines, growth factors and lipid mediators^54^, ^55^. The predominant immune cell population in this environment consists of macrophages; mainly tissue-resident macrophages and infiltrating recruited macrophages from bone marrow-derived monocytes^56^. These cells can be converted into TAMs through soluble factors present in the tumor microenvironment such as IL-6, interleukin-10 (IL-10), interleukin-4 (IL-4), interleukin-13 (IL-13) and TGF-β^57^-^60^.

TAMs are generally considered to be immunosuppressive cells that can promote tumor phenotypes and thus facilitate tumor growth and progression 61. They constitute the primary immune cell population within the ovarian cancer microenvironment, showing two main phenotypes depending on stimulus: anti‑tumorigenic macrophages M1-like or pro‑tumorigenic macrophages M2-like^62^. However, within this environment, TAMs are often polarized towards immunosuppressive M2-like phenotypes with high expression levels for scavenger receptor class B (CD163), mannose receptor (CD204), IL-10, chemokines CCL18 or CCL22^63^.

Ovarian cancer-derived exosomes are rich in various miRNAs and have been found to play an essential role in converting an M2 like phenotype. For example, EOC derived exosome miR-222-3p has been shown to promote TAMs polarization to inhibit anti-tumor immunity via activation of the signal transducer and activator of transcription 3 (STAT3) pathway^64^ , while elevated miR-200b levels in the plasma of ovarian cancer patients have been reported to promote macrophage M2 polarization but inhibit M1 polarization^65^, with higher expression levels enhancing proliferation and invasion of tumor cells.

TAMs have been found to affect both tumor cells and other immune cells in order to achieve tumor escape. Zhu et al. discovered that exosomes derived from macrophages under hypoxia and enriched with miR-223 could promote the malignant phenotype of EOC through the PTEN-PI3K/AKT pathway^66^. Additionally, TAMs-derived exosomes containing miR-29a-3p and miR-21-5p could inhibit STAT3, regulate Treg/Th17 cells, create an imbalance of these cell populations and ultimately generate an immune suppressive microenvironment that facilitates EOC progression and metastasis^67^.

### Relationship between Hypoxia and Exosomes

During peritoneal metastasis of ovarian cancer, tumor cells detach from the primary site to form single cells or multicellular tumorspheres into the peritoneal cavity. In this process, tumor cells are exposed to hypoxic conditions due to the inability to obtain a vascular supply 68. Hypoxic conditions can lead to the acquisition of a more aggressive malignant phenotype by tumor cells, allowing them to survive and colonize the peritoneal cavity to form metastases^69^, ^70^. Research on ovarian cancer has provided additional evidence to support the idea that hypoxia can trigger an increase in exosome production. Studies have shown that hypoxia can prompt ovarian cancer cells to release more exosomes, which in turn promote a more aggressive cancer phenotype and contribute to chemoresistance through the activation of STAT3/Rab proteins 71. These findings suggest that exosomes may play a crucial role in driving changes in tumor cells within a hypoxic microenvironment (Fig. 2C).

Notably, exosomes secreted by hypoxic ovarian cancer cells have been found to act on both ovarian cancer cells and other cells in the tumor microenvironment, such as CAFs and TAMs. For instance, high expression of miR‑940 in exosomes derived from EOC cells could induce macrophages to differentiate into the M2 phenotype, thereby promoting the proliferation and metastasis of EOC 72. Furthermore, Chen et al. demonstrated that exosomes secreted by hypoxic EOC cells can induce M2 macrophage polarization, thus promoting the proliferation and migration of EOC. Moreover, hypoxia-inducible factors were found to be crucial in this process. Microarray analysis of normoxic and hypoxic exosomes revealed that miR-21-3p, miR-125b-5p, and miR-181d-5p were enriched in hypoxic exosomes, which may be the key mediators of the tumor-promoting phenotype 73.

### Relationship between exosomes and blood-borne metastatic ovarian cancer

**Figure 3:**
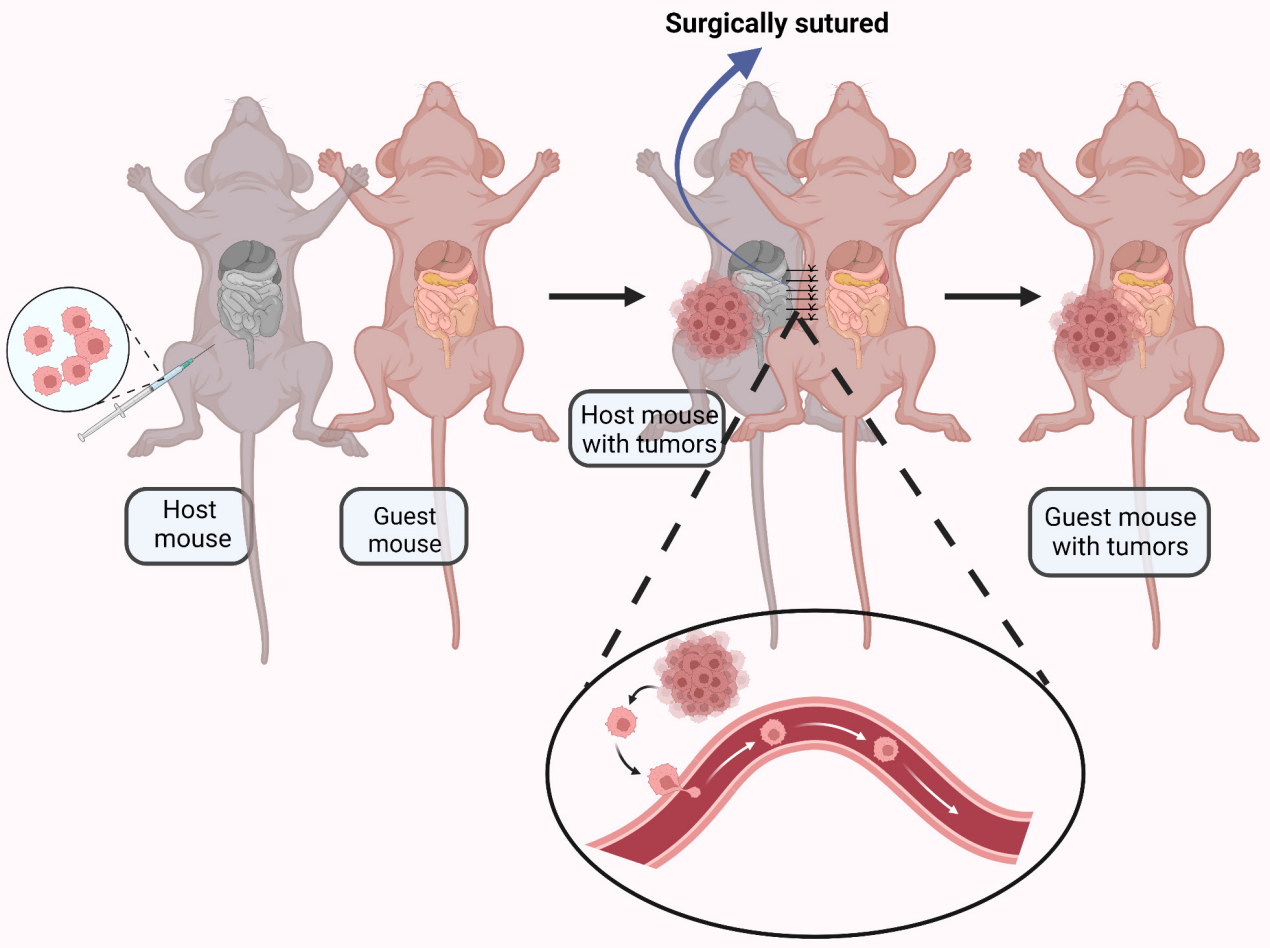
An ovarian cancer-bearing mouse and a healthy mouse were anastomosed together by surgical suturing. The ovarian cancer cells were transferred to the healthy mouse through the blood vessels connected between the mouse. Created with BioRender.com.

It is generally accepted that ovarian cancer metastasis results from direct surface spread and dissemination of detached tumor cells into the peritoneal cavity^74^. However, CTCs have observed in the blood of ovarian cancer patients 75, 76, and a significant number of ovarian cancer patients have distant metastasis at initial diagnosis 77, suggesting hematogenous metastasis may be possible. To explore this phenomenon, a parabiosis model was used to analyze underlying mechanisms of ovarian cancer spread^78^. A mouse with ovarian cancer was surgically anastomosed with a guest mouse without cancer. Metastasize to the omentum was found in the guest mouse, indicating hematogenous metastasis (Fig. 3). The paired mice shared blood but not lymphatic vessels, providing evidence for alternative strategies for prevention and treatment of ovarian cancer metastasis.

Exosome-derived cargoes are a critical factor in tumor metastasis. Exosome-derived cargoes are known to play an important role in tumor metastasis. Several studies have investigated crucial biomolecules carried by circulating exosomes in ovarian cancer plasma^79^, ^80^. A study showed that circulating exosomal circular forkhead box protein P1 (circFoxp1) was positively associated with distant metastasis, residual tumor diameter, and clinical response^81^. These findings suggest that hematogenous metastasis to the greater omentum may be as likely as direct dissemination in some cases—a potential mode of spread often overlooked.

## Clinical application of exosomes in ovarian cancer

### Exosomes in early diagnosis of ovarian cancer

At the initial diagnosis, most ovarian cancer patients have already developed peritoneal metastases^3^. Cancer metastasis is a lengthy process long before the second tumor engraftment, and exosomes play a critical role in this process. Therefore, exosome detection may offer the possibility of early diagnosis. Compared with non-exosomal detection methods, exosomes possess unique advantages such as their stable biological structure that prevents cargoes from RNase degradation. Numerous studies have demonstrated the potential of exosomes as biomarkers for ovarian metastasis (Table 1). For instance, CD24 and epithelial cell adhesion molecule (EpCAM) were found to be highly expressed in tumor-derived exosomes and were thus associated with ovarian cancer development^55^. Moreover, all samples tested positive for CD24 in ascites-derived exosomes even when the corresponding tumors were negative, which suggests that the ascites exosome test could be a sensitive method for diagnosing ovarian cancer. A National Cancer Institute (NCI-60) study further revealed that only 213 proteins were shared among 60 tumor cell lines from 9 different cancers 82, implying that exosome-specific proteins could potentially serve as tumor markers. In an effort to identify potential biomarkers of EOC, a cohort study used liquid chromatography tandem mass spectrometry (LC-MS/MS) with tandem mass tagging (TMT) to perform a comprehensive proteomic analysis of patient plasma derived from ovarian cancer-associated exosomes. They identified 50 genes which exhibited differential expression between EOC patients and those with benign diseases; bioinformatics analysis eventually selected GSN, FGG, FGA and LBP as potential diagnostic and prognostic biomarkers for EOC^83^. Additionally, miRNAs extracted from circulating exosomes have been explored as diagnostic markers for ovarian cancer since 2008^84^, with increasing number of studies being conducted on this topic over recent years^85^-^87^. For example, miR-99a-5p was significantly increased in microarray analysis of ovarian cancer derived exosomes; serum miR-99a levels in patients suffering from this disease were 1.7 and 2.8 times higher than those observed in benign tumor patients or healthy individuals respectively with high specificity (0.75) and sensitivity (0.85), thereby indicating its utility as a potential marker for early diagnosis of ovarian cancer^88^. Taken together, these findings point to the potential clinical value of utilizing exosomes for early diagnosis of ovarian cancer despite current limitations regarding extraction methods or detection techniques thereof. Nonetheless its attractive promise remains intact making it an option moving forward into clinical application.

### Exosomes in the treatment of ovarian cancer

#### Exosomes as therapeutic targets

Tumor-derived exosomes have been implicated in promoting tumor progression and drug resistance 10, 89. Recently, research on their potential therapeutic applications has become a hot topic. For instance, GW4869, an inhibitor of exosome release, was found to attenuate CD44 expression and reverse EMT in PMCs by inhibiting exosome release in ovarian cancer cells, thereby inhibiting ovarian cancer invasion 13. In another study, researchers utilized GW4869 treatment to inhibit miR-205 uptake by human umbilical vein endothelial cells (HUVECs), which effectively suppressed the invasion and angiogenesis of ovarian cancer. The same effect could also be achieved by filipin (an inhibitor of lipid raft-dependent and caveolar endocytosis) 11. Furthermore, stromal-derived exosomal miR-21 has been shown to promote paclitaxel resistance in ovarian cancer cells through APF1 90. These findings suggest that targeting and inhibiting exosome-derived miRNAs may serve as an adjuvant therapy for treating metastasis and drug resistance in ovarian cancer.

#### Exosomes as Drug Carriers

Exosomes possess several advantages, such as high tolerance, long circulation half-life, low toxicity, low immunogenicity, and inherent homing ability 91. Moreover, they can be genetically engineered to cross biological barriers such as the blood-brain barrier and carry a variety of drug molecules; thus preventing drug degradation 92. Exosomal formulations of antineoplastic agents have already been tested. For instance, exosomes containing triptolide were found to inhibit ovarian cancer cell proliferation as well as tumor growth and have good therapeutic potential 93. Additionally, berry anthocyanins encapsulated with exosomes as carriers exhibited promising therapeutic effects on both drug-sensitive and drug-resistant ovarian cancer cells; when combined with cisplatin it synergistically improved therapeutic activity 94. Furthermore, exosomal formulations of paclitaxel (PAC) for oral delivery can significantly reduce the side effects associated with PAC. Nie et al. used a chemical engineering approach to construct a responsive exosome nano-bioconjugate that cooperated with tumor immunotherapy 95. They conjugated azide-modified M1 macrophage-derived exosomes with dibenzocyclooctynes-modified antibodies of CD47 and SIRPα through pH-sensitive linker. Notably, in their experiment, BALB/c mice injected with 4T1 tumor cells were randomly divided into four groups: 1) PBS control, 2) free antibody, 3) M1 exosome, and 4) exosome nano-bioconjugates. All mice in forth group survived throughout the observation period without metastasis, whereas all those in the PBS group died within 24 days. Numerous studies have highlighted the potential value of exosomes as drug carriers for treating ovarian cancer (summarized in Table 2). However, their massive acquisition and engineering remain major challenges that need to be addressed.

## Summary

Metastasis of ovarian cancer is a complex process driven by various factors, including matrix remodeling, immunosuppression, angiogenesis, and changes in the tumor microenvironment. Recent studies have shed light on the molecular mechanisms underlying ovarian cancer metastasis and have made significant progress in exploring the role of exosomes in regulating this process. Exosomes offer new possibilities for early diagnosis and treatment of ovarian cancer through targeted medication, which can improve drug efficacy while reducing toxicity. Significant strides have been made in the application of engineered exosomes for the treatment of ovarian cancer. These remarkable achievements are a testament to the potential of exosomes as a therapeutic tool in the fight against this deadly disease. However, challenges still remain in applying exosomes to clinical practice due to limitations in detection and extraction methods. Further exploration into these issues is necessary to enable better clinical use of exosomes for diagnosis and treatment of ovarian cancer.

## Figures and Tables

**Table 1:** Potential exosomal biomarkers of diagnosis of ovarian cancer.

Type of factors	Factors	Source	Subtypes	Reference
protein	GSN, FGG, FGA and LBP	Plasma	EOCs	83
protein	CD24 EpCAM	Ascites	NA	55
miRNA	miR-99a-5p	Serum	EOCs	88
miRNA	miR-145, miR-200c, miR-21 and miR-93	Serum	EOCs	85
miRNA	miR-21, miR-100, miR-200b, miR-320, miR-16, miR-93, miR-126 and miR-223	Plasma	EOCs	86
miRNA	miR-21, miR-141, miR-200a/b/c, miR-203, miR-205 and miR-214	Serum	HGSC	84
miRNA	miR-200a, miR-200b and miR-200c	Serum	EOCs	87

Abbreviations: NA: not available; miRNA: microRNA; HGSC: high-grade serous carcinoma; EOC: epithelial ovarian cancer.

**Table 2:** Role of exosomes as drug carriers in ovarian cancer.

Source of exosomes	Loading approach	Cargo	Role	Reference
SKOV3	Sonication	Triptolide	Reduce the cytotoxic and apototic effects on SKOV3 cells. Enhance the inhibition of proliferation and growth.	93
Milk	Incubation	Berry anthocyanidins	Enhance the therapeutic activity of paclitaxel in vivo	94
Milk	Incubation	Cisplatin	Enhance the anti-cancer effect through avoiding endosome trapping	96
Macrophages from umbilical cord blood	Sonication	Cisplatin	Enhance the cytotoxicity in drug-resistant A2780/DDP cells and drug-sensitive A2780 cells.	97
Fibroblasts	Electroporation	miR-199a-3p	Suppressed c-Met expression, inhibited cell proliferation and invasion	98
